# The neuroprotective effect of Chinese herbal medicine for cerebral ischemia reperfusion injury through regulating mitophagy

**DOI:** 10.3389/fphar.2024.1378358

**Published:** 2024-06-04

**Authors:** Yanling Chen, Yanan Zhang, Qin Wu, Jing Chen, Yihui Deng

**Affiliations:** ^1^ School of Integrated Chinese and Western Medicine, Hunan University of Chinese Medicine, Changsha, China; ^2^ Hunan Province Key Laboratory of Cerebrovascular Disease Prevention and Treatment of Integrated Traditional Chinese and Western Medicine, Hunan University of Chinese Medicine, Changsha, China; ^3^ School of Traditional Chinese Medicine, Hunan University of Chinese Medicine, Changsha, China

**Keywords:** mitophagy, Chinese herbal medicine, cerebral ischemia reperfusion injury, neuroprotective effect, mitochondrial dynamics

## Abstract

The incidence of ischemic stroke has been increasing annually with an unfavorable prognosis. Cerebral ischemia reperfusion injury can exacerbate nerve damage. Effective mitochondrial quality control including mitochondrial fission, fusion and autophagy, is crucial for maintaining cellular homeostasis. Several studies have revealed the critical role of mitophagy in Cerebral ischemia reperfusion injury. Cerebral ischemia and hypoxia induce mitophagy, and mitophagy exhibits positive and negative effects in cerebral ischemia reperfusion injury. Studies have shown that Chinese herbal medicine can alleviate Cerebral ischemia reperfusion injury and serve as a neuroprotective agent by inhibiting or promoting mitophagy-mediated pathways. This review focuses on the mitochondrial dynamics and mitophagy-related pathways, as well as the role of mitophagy in ischemia reperfusion injury. Additionally, it discusses the therapeutic potential and benefits of Chinese herbal monomers and decoctions in the treatment of ischemic stroke.

## 1 Introduction

Stroke is the most common serious manifestation of cerebrovascular disease (Feske, 2021). Ischemic stroke accounts for the largest proportion of all types of stroke and has high mortality and severe disability rates worldwide (Kim et al., 2020; Thayabaranathan et al., 2022; Tsao et al., 2023). The main lesion of ischemic stroke is cerebral infarction, which causes clinical classic symptoms such as facial droop, weakness or numbness in arm, difficulty speaking, confusion, loss of vision and poor balance (Walter, 2022). The brain is the most sensitive of all human organs to ischemia. Insufficient blood supply to cerebral tissue, combined with hypoxia and glucose deprivation, results in irreversible impairment of neuronal function and tissue integrity (Feske, 2021). The ultimate goal of therapeutic intervention for ischemic stroke is to restore sufficient blood flow to the damaged tissue. The common modern treatments include surgery, thrombolysis and thrombectomy (Herpich and Rincon, 2020).

The process of restoring blood flow after cerebral ischemia and inducing tissue reperfusion injury is commonly known as cerebral ischemia/reperfusion (I/R) injury (Kalogeris et al., 2016). The pathological mechanisms of cerebral I/R injury are complex, including oxidative stress, ion balance disturbance, inflammation responses, mitochondrial energy metabolism disturbance, mitophagy, apoptosis, blood-brain barrier destruction and aggravation of capillary no-reflow (Kalogeris et al., 2016; Jiang et al., 2018; Wang et al., 2018; Wu et al., 2018; Malone et al., 2019). Mitochondrial autophagy, also referred to as mitophagy, is a type of autophagy that continually monitors the quality of mitochondria and eliminates damaged mitochondria through lysosomal targeting via various pathways (Ashrafi and Schwarz, 2013). The role of mitophagy in cerebral I/R remains controversial. Inadequate removal of damaged mitochondria or excessive degradation of essential mitochondria both lead to cell death (De et al., 2021). The prevailing opinion is that the activation of autophagy mitigates cerebral I/R injury through multiple pathways, such as reducing damage to neurons, glia, and endothelial cells (Papadakis et al., 2013). Additionally, mitophagy provides neuroprotection by inhibiting inflammasome activation (Zhou et al., 2011; Zhong et al., 2016a).

Chinese herbal medicine (CHM) has been used for centuries to treat ischemic stroke, with proven clinical efficacy (Li et al., 2022). Animal and cellular experiments have further demonstrated that CHM monomers and decoctions can improve ischemic stroke symptoms and provide neuroprotection by attenuating cerebral I/R injury (Ding et al., 2019; Jiao-Yan et al., 2021; Tang et al., 2021; Ni et al., 2022; Liao et al., 2023). These studies have found that many Chinese medicines, including monomers and decoctions, can attenuate cerebral I/R injury by modulating mitophagy (Zhang et al., 2020a; Han et al., 2022a; Ji et al., 2022a; Huang et al., 2023a). Interestingly, some of these medicines work by inhibiting mitophagy, while others work by activating it. The ameliorative effects of these herbs on ischemic stroke are consistent, regardless of whether they inhibit or activate mitophagy. Furthermore, some of these herbs are clinically used together in one herbal formula to treat ischemic stroke (Ji et al., 2022a). There is limited literature on this interesting phenomenon, and the exact reasons for it are unclear. However, it demonstrates the potential and advantages of herbal medicine in treating ischemic stroke by mediating mitophagy.

The current study briefly introduced the physiological functions of mitochondria and the molecular mechanism of mitophagy, further explaining the complex pathological mechanism of mitophagy involved in cerebral I/R. We summarised the existing evidence on Chinese herbal medicine targeting mitochondrial autophagy to improve cerebral ischemic reperfusion. We also discussed the potential therapeutic value of Chinese herbal medicine for cerebral ischemic stroke. This may provide new directions for future research.

## 2 Mitochondria and mitophagy

### 2.1 Mitochondrial dynamics and mitochondrial quality control

Mitochondria are crucial organelles for energy metabolism in the human body. They play a vital role in ATP production, phospholipid biosynthesis and transport, calcium signaling and iron homeostasis (Friedman and Nunnari, 2014). Mitochondrial dysfunction can disrupt cell homeostasis, which is the pathological basis of many diseases, including cardiovascular diseases, neurodegenerative diseases and cancers. Mitochondria maintain their quality control and dynamic balance through continuous fission, fusion, motility and autophagy, which are regulated by multiple mechanisms.

**FIGURE 1:**
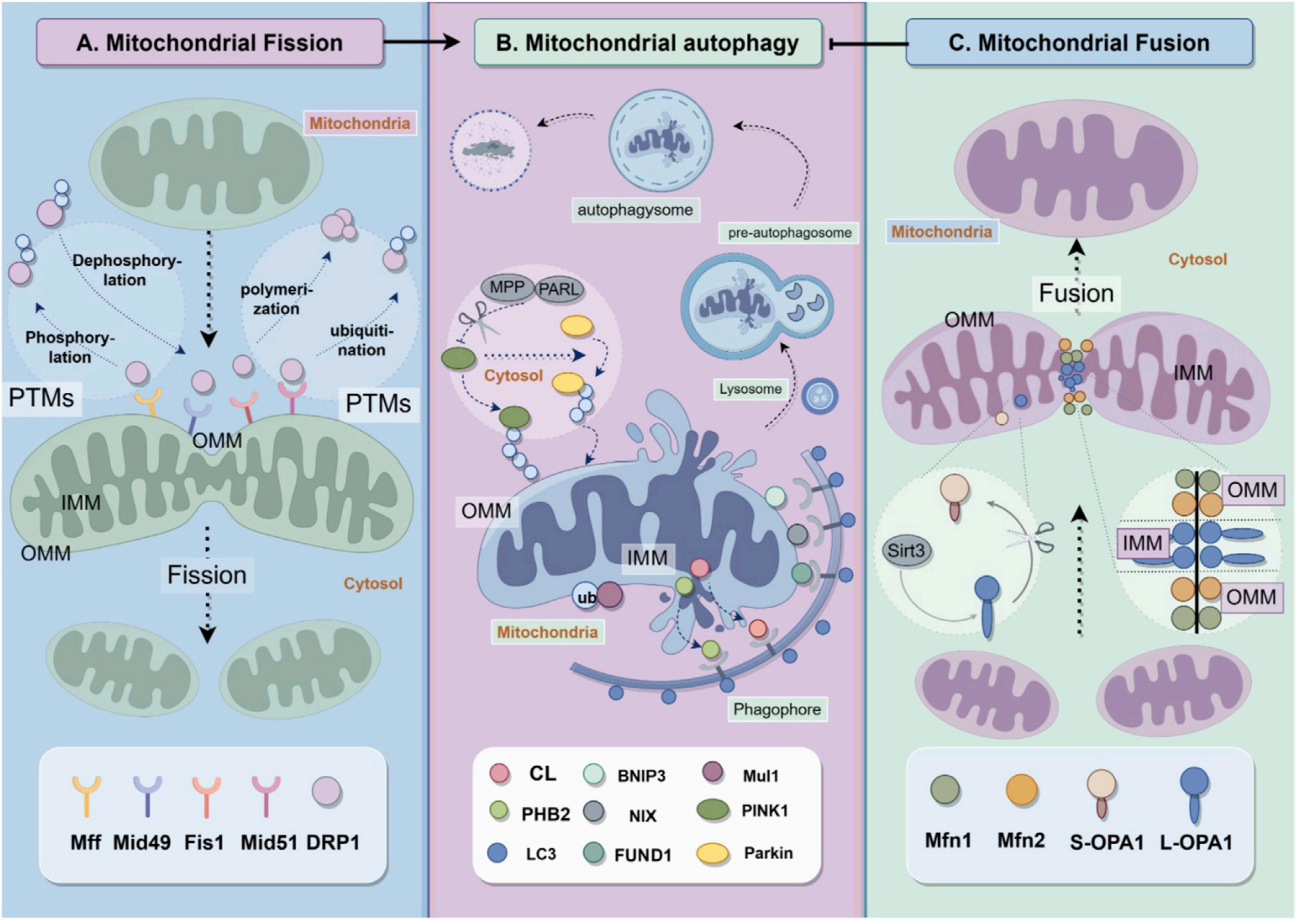
Mitochondrial quality control includes fission, fusion and autophagy. **(A)** Mitochondrial fission depends on the recruitment of Drp1 from cytosol to mitochondria to react with Drp1 receptor proteins Mff, Mid49, Fis1 and Mid51. **(B)** Mitochondrial autphagy requires various pathways, including PINK1/Parkin, Mul1/Mfn2 pathways and receptor dependent pathways. The proteins on outer mitochondrial membranes and inner mitochondrial membranes play vital role in the formation of autophagysomes. **(C)** mitochondrial fusion is regulated by dynamin-related proteins including Mfn1, Mfn2 and OPA1.

Mitochondrial fission is primarily mediated by dynamin-related protein 1 (Drp1). Drp1 is a large GTPase, and also belongs to the mitochondrial dynamin family. Drp1 is located in the cytoplasm and can be recruited to the outer mitochondrial membrane to interact with the four fission proteins involved in this mechanism (Westermann, 2010): mitochondrial fission factor (Mff) (Otera et al., 2010), mitochondrial dynamics protein of 49 kDa (Mid49) (Palmer et al., 2011), Mid51 (Palmer et al., 2011) and fission1 (Fis1) (Zhang and Hu, 2008). The combination of Drp1 and fission proteins constricts mitochondria, resulting in the division of mitochondria into separate entities (Figure 1A). An increasing number of studies suggest that post-translational modifications (PTMs) of Drp1 and fission proteins, such as phosphorylation, dephosphorylation, ubiquitination, and polymerization, are crucial in regulating mitochondrial fission (Yamada et al., 2018; Yu et al., 2020; Kleele et al., 2021; Yu et al., 2021). Mitochondria that have undergone mitochondrial fission become smaller and more susceptible to mitophagy.

Mitochondrial fusion is also primarily regulated by dynamin-related proteins, as shown in Figure 1C. Mitochondria are organelles with double membranes, consisting of outer and inner membranes. Mitofusin1 (Mfn1) and mitofusin2 (Mfn2) are proteins located in the outer mitochondrial membrane (OMM) that mediate OMM fusion, while Optic atrophy 1 (OPA1) is a protein located in the inner mitochondrial membrane (IMM) that mediates IMM fusion. Downregulation of mitochondrial fusion improves mitophagy (Archer, 2013).

Mitochondrial motility is linked to mitochondrial dynamics. Mitochondria are in constant motion along cytoskeletal tracks that contain motility proteins, dynein and kinesin. The OMM protein Miro combined motility proteins through milton. Research has shown that the phosphorylation and degradation of Miro can arrest mitochondrial motility (Wang et al., 2011).

Both mitochondrial fission and fusion are important quality control mechanisms that regulate mitophagy and ensure mitochondrial function. Fission separates depolarized mitochondria from the network, and a coordinated downregulation of fusion mediators prevents the re-integration of these mitochondria, which then triggers mitophagy. Additionally, the motility of mitochondria is also involved in mitophagy.

Mitophagy is a type of autophagy that selectively eliminates mitochondria. Cellular autophagy, also known as type II programmed cell death, refers to the self-digestive process in which cells use lysosomes to degrade their own damaged, denatured, or aged macromolecules and organelles under the influence of external environmental factors (Dikic, 2017). Autophagy is initiated in response to stimuli such as hypoxia, starvation, and calcium overload. This process involves the formation of a double-membrane cup-like dividing membrane around the material to be degraded, which then forms a pre-autophagosome. The divided membrane gradually extends until the degraded material is completely enclosed, forming the autophagosome. Autophagosomes merge with lysosomes to form autophagic lysosomes, which are eventually fully degraded and absorbed (Palikaras et al., 2018) (Figure 1B).

### 2.2 Signaling pathways

#### 2.2.1 Receptor-independent mitophagy

##### 2.2.1.1 PINK1/parkin

Since the ubiquitin-proteasome system plays an important role in mitophagy by degrading mitochondrial outer membrane proteins, the synergistic interaction between the Ser/Thr kinase PINK1 and the E3 ubiquitin ligase Parkin is one of the most important signaling pathways for mitophagy and has been the most studied (Wen et al., 2021). In normal cells, PINK1 remains inactive in the cytosol due to the coordination between the mitochondrial processing peptidase (MPP) and the rhomboid-like serine protease PARL (Deas et al., 2011; Greene et al., 2012). Mitochondria dysfunctions, such as the dissipation of mitochondrial membrane potential (MMP), inhibit the MPP/PARL-mediated process and promote the homodimerization and autophosphorylation of PINK1. This, in turn, activate PINK1 and facilitates the translocation of Parkin to mitochondria (Okatsu et al., 2012; Aerts et al., 2015). PINK1 phosphorylates Parkin S65 in the UBL domain and also phosphorylates mitochondrial ubiquitin chains to promote Parkin activation for mitophagy both directly and indirectly (Koyano et al., 2014). The ubiquitination and phosphorylation of multiple OMM proteins lead to a feed-forward signaling for mitophagy adapter proteins which link the polyubiquitin signal with LC-3, resulting in the formation of autophagosomes for mitochondria.

Recent studies have shown that deubiquitinases, such as USP8, USP15 and USP30, play a crucial role in PINK1/Parkin-mediated mitophagy. While USP8 promotes mitophagy (Durcan et al., 2014), USP15 and USP30 remove the ubiquitin chains from mitochondrial proteins, thereby inhibiting mitophagy (Bingol et al., 2014; Cornelissen et al., 2014).

##### 2.2.1.2 Mul1/Mfn2

Mitochondrial ubiquitin ligase 1 (Mul1) is a protein located in mitochondrial membrane that triggers trigger mitophagy by promoting the ubiquitination and degradation of the mitochondrial fusion protein mitifusion1 (Mfn2). In skeletal muscles and myoblast cultures, upregulation of Mul1 promoted mitophagy by inhibiting Mfn2 (Lokireddy et al., 2012). Mul1 deficiency increased Mfn2 activity and suppressed neuronal mitophagy (Puri et al., 2020). The Mul/Mfn2 pathway that regulates mitophagy is thought to be Parkin-independent.

#### 2.2.2 Receptor-dependent mitophagy

Several mitophagy receptor proteins, such as FUNDC1, BNIP3, NIX and CL, are located on the mitochondrial membrane. These proteins, including inner and outer membrane receptor proteins can directly interact with LC-3B and promote mitochondrial autophagy by recruiting autophagosomes to damaged mitochondria (Lampert et al., 2019).

##### 2.2.2.1 Outer membrane protein receptors

BCL2 and adenovirus E1B 19-kDa-interacting protein3 (BNIP3) and BNIP3-like (BNIP3L), also known as NIX, are integral outer mitochondrial membrane proteins. During hypoxia, BNIP3 is upregulated, homodimerizes and binds to LC3 to induce mitophagy. BNIP3L shares 53%–56% amino acid sequence homology with BNIP3 (Zhang and Ney, 2009). Under hypoxic conditions, BNIP3 and NIX are activated at the mRNA level in an HIF-1α-dependent manner. BNIP3 binds and inhibits the activity of Rheb, a mammalian Sirolimus target protein activator critical for mTORC1 activation, which negatively regulates bulk autophagy and mitophagy. Therefore, BNIP3 can activate mitophagy (Novak et al., 2010). FUNDC1 is also an integral OMM protein that functions as a receptor for mitophagy through its dephosphorylation (Liu et al., 2012). Dephosphorylated FUNDC1 has greater binding affinity for LC3 than NIX. Bcl-2-like protein 13 (BCL2L13) is a single-pass membrane protein anchored to the outer mitochondrial membrane. Its function is to regulate mitochondrial morphology and interacts with ULK1 to localize the mitophagy initiation complex to mitochondria (Murakawa et al., 2015; Murakawa et al., 2019).

##### 2.2.2.2 Inner membrane protein receptors

Cardiolipin (CL) is a phospholipid dimer synthesized in the inner mitochondrial membrane (IMM). It externalises to the mitochondrial outer surface to interact with mitophagy proteins and initiate mitophagy in response to mitochondrial injury and depolarisation (Chu et al., 2013).

PHB2 is an IMM mitophagy receptor. Proteasomal-dependent OMM rupture is necessary for PHB2 before it binds to LC3 II. The LIR Domain of PHB2 is required for Parkin-mediated mitophagy (Wei et al., 2017b). The mechanisms of mitophagy is shown in Figure 1B.

## 3 The role of mitophagy in the pathological mechanism of cerebral I/R injury

### 3.1 Ischemia and hypoxia lead to mitochondrial dysfunction and induce mitophagy

The brain requires a significant amount of ATP to maintain its function in the human body. Mitochondria are the primary source of ATP production, resulting in higher mitochondrial content in brain tissue than in other tissues (Tang et al., 2016). However, since the energy storage in brain tissue is lower compared to other organs in the body, even a transient ischemia-hypoxia state can cause severe and potentially irreversible damage to brain tissue cells (Tierradentro-García et al., 2023). When brain tissue cells transition from normoxia to ischemia-hypoxia due to arterial blood flow obstruction, cellular anaerobic metabolism is induced. This impairs mitochondrial ATP synthesis, causes dysfunction in the mitochondrial electron transport chain, and significantly dissipates the mitochondrial membrane potential. These changes affect the operation of cellular ion exchange channels. As a result, Ca2+, sodium and hydrogen ions accumulate in the cell, leading to hyperosmolarity and causing the cell to swell to a certain degree. Additionally, cytoplasmic enzyme activity is impaired (Hofmeijer and van Putten, 2012; Dharmasaroja, 2016). The mitochondria must take up a large amount of Ca2+, which causes them to swell and become impaired. To maintain cellular homeostasis, the cell must engulf the damaged mitochondria (Lampert et al., 2019). Furthermore, during ischemic hypoxia, intracellular antioxidant levels decrease, leading to an overproduction of reactive oxygen species (ROS) and promoting oxidative stress (Yingze et al., 2022). mitophagy is induced as a means to reduce ROS levels (Shadel and Horvath, 2015). In summary, ischemia and hypoxia result in mitochondrial dysfunction and induce mitophagy through various mechanisms.

### 3.2 Mitophagy has dual roles in cerebral I/R injury

Mitophagy plays a dual role in cerebral ischemia reperfusion injury, and the mechanism remains complex and controversial.

During the ischemic phase, pathological factors induce mitochondrial dysfunction. The mitochondrial damage is a significant contributor to the production of oxidative stress and can lead to cell death (Granger and Kvietys, 2015). Restoring the blood flow during the reperfusion stage brings fresh oxygen but also leads to a high production of ROS. Mitophagy is a form of selective autophagy that eliminates damaged mitochondria, attenuating cerebral I/R injury (Baek et al., 2014a). Appropriate mitophagy promotes the restoration of intracellular homeostasis to a certain extent, thus providing neuroprotection. Promoting mitophagy during the rapid phase of reperfusion favours neuronal survival (Zhang, 2013; Liu et al., 2014; Yuan et al., 2017). It has been found that activation of PINK1/Parkin-dependent mitophagy ameliorates neuronal damage in the cortex and hippocampal CA1 region after cerebral ischemia and removes damaged mitochondria (Wang and Zhou, 2020; Wu et al., 2021). The neuroprotective effect of tPA on CIRI can be achieved by promoting FUNDC1-dependent mitophagy, which improves mitochondrial function and inhibits apoptosis (Cai et al., 2021). It has been found that promotion of mitochondrial autophagy through PINK/PARKIN-mediated ubiquitination of PA2G4/EBP1 contributes to neuroprotection after CIRI (Hwang et al., 2024).

However, if the stress caused by reperfusion is too high and autophagy fails to relieve it, then cellular damage will still occur (Qin et al., 2016). Excessive mitophagy can also induce cell death and aggravate brain injury. The reperfusion of ischemic cerebral tissues changes dynamically. Recent studies have shown that mitophagy may have varying effects on neurons in response to ischemia and during different pathological stages of reperfusion. In the early stage of CIRI, mitophagy can engulf a large number of functionally impaired mitochondria, recycle the useful substances in them and synthesize new mitochondria. The activation of mitophagy facilitates the restoration of the balance of energy metabolism in the brain. In the middle and late stages of CIRI, excessive mitophagy will lead to a shortage of mitochondria, which is not conducive to the recovery of brain injury (Baek et al., 2014b; Zhong et al., 2016b; Sulkshane et al., 2021). However, the reasons for this change have not been clearly investigated.

## 4 Regulation for management of cerebral I/R injury

CHM are one of the common therapies used clinically for the treatment of ischemic stroke in China. Natural compounds have also gained the attention of many researchers in recent years. By summarising some current *in vitro* and *in vivo* studies of CHM, we explored the mechanism by which these herbs act on mitophagy and thus alleviate CIRI. Moreover, CHM could regulate some important pathological aspects of CIRI, such as calcium overload, inflammation, apoptosis and oxidative stress through mitophagy. These interesting phenomena may bring some inspiration for subsequent studies.

### 4.1 Monomers/decoctions promoting mitophagy for cerebral I/R injury

**Ligustilide (LIG)** is a natural monomer isolated from *Ligusticum chuanxiong hort* and *Angelica sinensis* (Oliv.) *diels*. Both of them have the effect of activating blood circulation and removing blood stasis in Chinese medicine. Recent studies have shown that LIG protects hippocampal neurons from CIRI both *in vivo and in vitro* (Gan et al., 2020; Sun et al., 2021). The research found that LIG reduced hippocampal neuron injury in both middle cerebral artery occlusion and reperfusion (MCAO/R) Sprague-Dawley (SD) rat models and oxygen-glucose deprivation and reoxygenation (OGD/R) HT-22 cell models. This was achieved by significantly elevating the protein expressions of PINK and Parkin (Mao et al., 2022), which enhance mitophagy. The neuroprotective effects of LIG were counteracted by midivi-1, a mitochondrial-autophagy inhibitor. Furthermore, the silencing of PINK1 partially impeded the effect of LIG on mitophagy. Therefore, it is possible that LIG can mitigate the ischemia/reperfusion injury by activating mitophagy through the PINK1/Parkin pathway.

***Panax notoginseng Saponins* (PNS)** is the main active ingredient extracted from the rhizome of *P. notoginseng (Burkill)*, a traditional Chinese medicine. *P. notoginseng* is widely used clinically to treat cardiovascular and cerebrovascular diseases (Tong et al., 2019). Mitophagy inhibits the activation of the NLRP3 inflammasome in cerebral ischemia (Shao et al., 2018; Mai et al., 2019; Ren et al., 2019; Xu et al., 2019). The inflammasome is central to the inflammatory response and mediates many neurodegenerative diseases (Prabhakaran et al., 2015; Mangan et al., 2018). PNS restrained NLRP3 inflammasome activation and activated mitochondrial autophagy via the PINK1/Parkin pathway in cerebral I/R injury rat brains (Xiao et al., 2022). Xuesaitong (XST), a Chinese medicinal preparation containing PNS, is widely used in the treatment of ischemic stroke. A study showed that it combined with dexmedetomidine I attenuated I/R injury by activating Keap1/Nrf2 signaling and mitophagy (Han et al., 2022b).

**Jionoside A1** is a native compound derived from *Rehmannia glutinosa* (Di-huang), a Chinese medicine that is commonly used to treat brain diseases, including stroke. *Rehmannia glutinosa* has been found to be neuroprotective against acute cerebral ischemia and to improve cognitive dysfunction (Fu et al., 2022; Wang et al., 2022). Although the study did not show significant alterations in Parkin and FUNDC1 expression, it did reveal that Jionoside A1 significantly enhanced mitochondrial content and promoted mitophagy in OGD/R and MCAO/R models by upregulating the expression of NIX protein in ischemic stroke I/R injury (Fu et al., 2022). Furthermore, Jionoside A1 was found to increase the cellular ATP levels and decrease the release of LDH, which is an indicator of neuronal cytotoxicity in the OGD/R model. Additionally, the knockdown of NIX inhibited the aforementioned effects of Jionoside A1 (Yu et al., 2023).

***Baicalein 7-O-β-D-glucuronide*** (**Baicalin)**, a natural flavonoid isolated from *Scutellaria baicalensis Georgi* (Huang-qin or Chinese skullcap), has been shown to provide neuron protection from cerebral ischemia by inhibiting inflammation, reducing apoptosis and regulating mitochondrial functions (Zhang et al., 2017; Hao et al., 2023). In the MCAO/R model, Baicalin decreased infarction volume and attenuated neurobehaviors (Li et al., 2017). Baicalin treatment inhibited the expression of Drp1 and promoted the synthesis of Mfn2, regulating mitochondrial fission and fusion. Additionally, it suppressed the production of ROS and elevated MMP in OGD/R PC12 cells through the activation of protein kinase by adenosine monophosphate (AMPK), resulting in suppressing cell apoptosis and enhancement of mitophagy (Li et al., 2017).

**Resveratrol (Res)** is a natural extract from *Rhizoma Polygoni Cuspidati* (Hu-zhang), a traditional Chinese medicine. It has an overall neuroprotective role in ischemic stroke (Hou et al., 2018; Ashafaq et al., 2021). Res activates autophagy by increasing the level of phosphorylated AMPK in cerebral ischemia rats and attenuates the mitochondrial failure in neuronal cultures (Pineda-Ramírez et al., 2020). According to a study, Res controlled the PINK1/Parkin-mediated mitophagy in OGD/R-injured neuron cells, increasing cell viability and suppressing apoptosis (Ye et al., 2021). The neuroprotective benefits of Res treatment were diminished due to the inhibition of mitophagy caused by the downregulation of PINK1 or Parkin.

**Taohong Siwu Decoction (THSWD)** is a compounded Chinese medicinal preparation used to treat blood stasis diseases, including stroke. The formula comprises six herbs: Tao-Ren [(*Prunus persica (L.) Batsch*], Dang-Gui [*A. sinensis (Oliv.) Diels*], Shu-Di-Huang [*R. glutinosa (Gaertn.) DC.*], Hong-Hua [*Carthamus tinctorius L.*], Chuan-Xiong [*Conioselinum anthriscoides*] and Bai-Shao [*Paeonia lactiflora Pall*]. THSWD has been found to alleviate cerebral I/R injury by suppressing cell pyroptosis (Wang et al., 2020), necrosis and neuroinflammation (Wang et al., 2021). It increased the expression of autophagy-related proteins (LC3 and Beclin1) and mitophagy marker proteins (PINK1 and Parkin), and reduced reactive oxygen species (ROS), the NLRP3 inflammasome and pro-inflammatory cytokines in OGD/R-induced P12 cells (Ji et al., 2022b; Shi et al., 2023). Furthermore, the formula also significantly reduced neurological deficit scores and cerebral infarct volume in MCAO/R rats (Shi et al., 2023). The effects of THSWD above were blocked with the use of Mdivi-1 both *in vivo and in vitro*.

**Dengzhan Xixin Injection (DX)** is a widely prescribed Chinese medicine injection derived mainly from Erigeron breviscapus (Vaniot) Hand.-Mazz. (National Pharmacopoeia Commission, 2020). It has been widely prescribed for years to treat cerebral ischemic stroke (Hu et al., 2020). The six components of DX, including scutellarin, 3,5-OdiCQA, 4,5-O-diCQA, 3,4-O-diCQA, caffeic acid and 5-O-CQA were determined using high performance liquid chromatography (HPLC). Scutellarin was the major representative compound with the highest concentration. DX administration attenuated cerebral infarction volumes and neuronal loss in rats and alleviated cerebral I/R injury by activating mitophagy. This was achieved via stimulation the protein expression level of LC3, PINK1 and Parkin (Yang et al., 2022). DX reduced ROS and MDA levels and increased SOD levels. DX intervention reversed the imbalance of ATP and MMP and maintained mitochondrial ultra structure by increasing the number of autophagic lysosomes. Interestingly, DX also decreased the expressions pf apoptosis-related proteins including Bax, Cyto-c and cleaved Caspase-3 and increased Bcl-2 level (Yang et al., 2022).

The table below summarises the studies on the promotion of mitophagy in cerebral I/R injury by traditional Chinese medicine (Table 1).

**TABLE 1:** Monomers/decoctions promoting mitophagy for cerebral IR injury.

Monomers/Decoctions	Source	*In vitro* model	*In vivo* model	Target	Other pathways	Chemical structure	References
Ligustilide (LIG)	*Ligusticum chuanxiong hort/Angelica sinensis (Oliv.) diels*	OGD/R-induced HT-22 cells	MCAO/R-induced	PINK1/Parkin	——	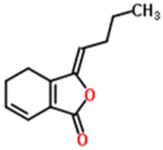	Mao et al. (2022)
			SD rats				
Panax notoginseng saponins (PNS)	*Panax notoginseng (Burkill)*	——	MCAO/R-induced	PINK1/Parkin	NLRP3	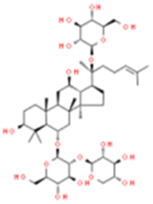	Xiao et al. (2022)
			SD rats				
Jionoside A1	*Rehmannia glutinosa*	OGD/R-induced cortex primary neuron cells	MCAO/R-induced	NIX	——	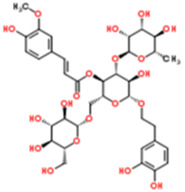	Yu et al. (2023)
			SD rats				
Baicalin	*Scutellaria baicalensis Georgi*	OGD/R-induced PC12 cells	MCAO/R-induced	Drp1/Mfn2	AMPK	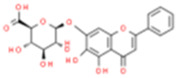	Li et al. (2017)
			SD rats				
Resveratrol (Res)	*Rhizoma Polygoni Cuspidati*	OGD/R-induced rat cortical neuron cells	——	PINK1/Parkin	——	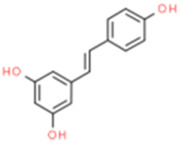	Ye et al. (2020)
Taohong Siwu Decoction (THSWD)	*Tao-Ren* [(*Prunus persica (L.) Batsch*]	OGD/R-induced PC12 cells	MCAO/R-induced SD rats	PINK1/Parkin	NLRP3	——	Ji et al. (2022) Shi et al. (2023)
	*Dang-Gui* [*Angelica sinensis (Oliv.) Diels*]						
	*Shu-Di-Huang* [*Rehmannia glutinosa (Gaertn.) DC.*],						
	*Hong-Hua [Carthamus tinctorius L.]*						
	*Chuan-Xiong [Conioselinum anthriscoides]*						
	*Bai-Shao [Paeonia lactiflora Pall]*						
Dengzhan Xixin injection	*Erigeron breviscapus (Vaniot) Hand.-Mazz*	——	MCAO/R-induced	PINK1/Parkin	——	——	Yang et al. (2022)
			SD rats				

**FIGURE 2:**
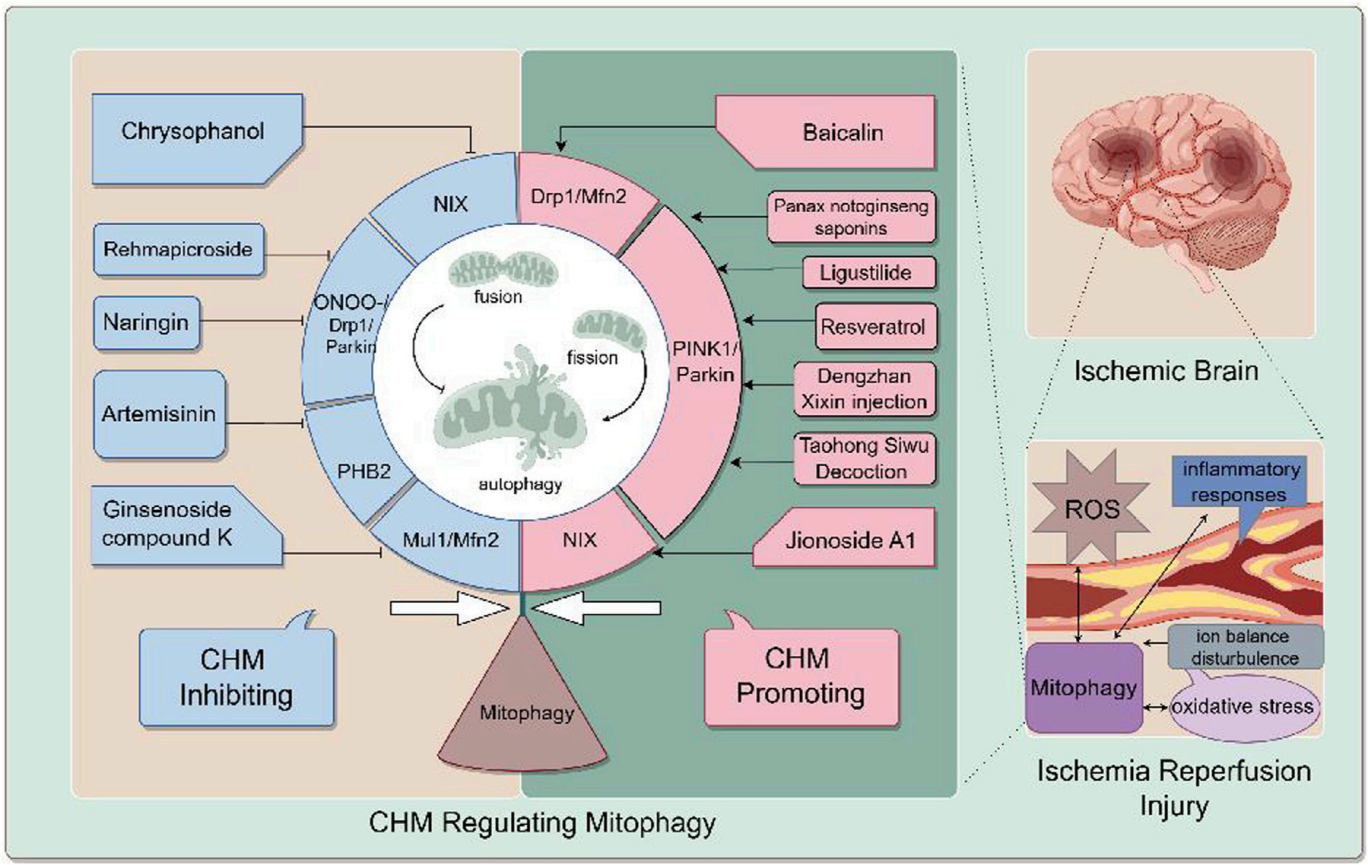
the mechanism of CIRI is complicated, including oxidative stress, ion balance disturbance, inflammation responses, blood-brain barrier destruction and mitophagy. CHM promoting mitophagy for cerebral IR injury include Ligustilide (LIG), Panax notoginseng saponins (PNS), Jionoside A1, Baicalin, Resveratrol (Res), Taohong Siwu Decoction (THSWD) and Dengzhan Xixin injection. CHM inhibiting mitophagy for cerebral IR injury include Ginsenoside compound K (CK), Rehmapicroside (Reh), Naringin, Artemisinin (ART), Chrysophanol (Chry), Xiao-Xu-Ming Decoction (XXMD). CHM regulates mitophagy to alleviate cerebral I/R inury through various pathways. For example, Ligustilide promotes mitophagy through the PINK1/Parkin pathway.

Figure 2 generally demonstrates the improvement of CHM for cerebral ischemia reperfusion injury by inhibiting or promoting mitophagy through different signaling pathways.

### 4.2 Monomers/decoctions inhibiting mitophagy for cerebral I/R injury

*Panax ginseng* is a Chinese herbal medicine that has been used as a tonic medicine for centuries in China. **Ginsenoside compound K(CK)**, an important saponin-like component of ginseng, augmented mitochondrial fusion and attenuated mitophagy in cerebral IR injury through the Mul1/Mfn2 pathway (Huang et al., 2023b). The study found that Ginsenoside CK inhibited mitochondrial dynamics imbalance and damage by reducing the binding affinity of Mul1 and Mfn2, which in turn inhibited the ubiquitination of Mfn2 by Mul1. Additionally, in OGD/R-induced PC12 cells, Ginsenoside CK was found to rescue mitochondrial dysfunction by promoting mitochondrial oxygen consumption. In the cerebral I/R injury rat model, the infarct volume of the pretreatment group was significantly smaller than that of control group. Ginsenoside CK reduced mitochondrial fission and mitophagy, counteracting I/R-induced neurological impairment and mitochondrial dysfunction. The mitigation effect of ginsenoside CK on mitophagy was markedly abrogated by Mfn2 knockdown (Huang et al., 2023b).

*Radix Rehmanniae,* a medicinal plant, is commonly used in Chinese medicine formulas for cerebral diseases (Zhou et al., 2016; Fei et al., 2018). **Rehmapicroside (Reh)**, a natural compound found in *Radix Rehmanniae*, has neuroprotective effects against cerebral IR injury by inhibiting peroxynitrite (ONOO−)-mediated mitophagy activation (Zhang et al., 2020b). During cerebral ischemia reperfusion injury, a large amount of NO and superoxide anion (O2−) contemporaneously form ONOO−. ONOO− is a cytotoxic factor that exacerbate neuronal damage by causing both oxidative and nitrosative stress, leading to cerebral impairments in I/R (Chen X. M. et al., 2013; Lourenco et al., 2017). It promotes DRP1 nitration and the recruitment of DRP one to impaired mitochondria, causing over-activation of mitophagy (Feng et al., 2017; Feng et al., 2018a; Li et al., 2019). The study found that Reh reacted with ONOO−, resulting in a decrease of ONOO−. Additionally, Reh downregulated PINK1, Parkin, p62 and the ratio of LC3-II to LC3-I in the OGD/RO-treated PC12 cells. Reh attenuated infarct size and improved neurological deficit scores in the MCAO rats by preventing the translocations of PINK1, Parkin and Drp1 into mitochondria for mitophagy. Furthermore, NADPH oxidases and iNOS expression were also suppressed by Reh (Zhang et al., 2020b).

**Naringin** (4′,5,7-trihydroxy-flavonone-7-rhamnoglucoside) is a bioflavonoid extracted from grapefruit and related citrus species, and has also been found in many herbal medicines for various diseases (Chen Y. et al., 2013; Li et al., 2014; Sharma et al., 2015). Recently, Naringin has shown potential in protecting cerebral neurons in different animal models including traumatic brain injury (Cui et al., 2014), subarachnoid hemorrhage (Han et al., 2016) and cerebral I/R injury (Gaur et al., 2009). Naringin reduced excessive mitophagy activation in the ischemic brains during cerebral I/R injury by scavenging ONOO− (Feng et al., 2018b). It also inhibited the production of superoxide and nitric oxide in SH-SY5Y cells under OGD/RO condition. Additionally, Naringin inhibited the expression of NADPH oxidase subunits and iNOS in rat brains with cerebral I/R injury. Naringin decreased the formation of 3-nitrotyrosine formation, inhibited the translocation of Parkin to the mitochondria and reduced the ratio of LC3-II to LC3-I in the mitochondrial fraction (Feng et al., 2018b).

**Artemisinin (ART)** is a bioactive compound derived from the plant *Artemisia annua*, known for its effectiveness in treating malaria (Tu, 2016). Studies showed that ART inhibited autophagy to alleviate apoptosis and oxidative stress in MPP(+)-induced SH-SY5Y cells (Yan et al., 2021), and it possessed the ability to reverse the oxidative stress damage aggravated by OGD/R treatment in the human neuroblastoma SH-SY5Y cells (Jiang et al., 2022). The antioxidative stress effect of ART is related to PHB2-mediated autophagy prohibitin 2 (PHB2). Unlike other autophagy receptors that target outer mitochondrial membrane (Lazarou et al., 2015), PHB2 is a mitophagy receptor on mitochondrial inner membrane that binds to LC3 and leads to mitopagy (Wei et al., 2017a). ART impaired mitophagy by inhibiting PHB2, which decreased the conversions of LC3I to LC3II. Silencing PHB2 eliminated the protective effect of ART against OGD/R-induced oxidative stress damage (Jiang et al., 2022). Other studies showed that ART also reduced the inflammation by activating Nrf2 and ROS-dependent p38 MAPK against cerebral ischemia reperfusion injury (Lu et al., 2018).

**Chrysophanol (Chry)** is the main active ingredient isolated from the rhizome of Da-Huang (*rhubarb*), a traditional Chinese medicine. Chry alleviated cerebral I/R injury in mice though inhibiting neuroinflammation and nitrosative/oxidative stress (Liu et al., 2022a; Liu et al., 2022b). A study found that Chry reduced mitophagy of hippocampus through inhibiting the expression of LC3B and NIX in bilateral common carotid arteries occlusion and reperfusion (CCAO/R)-induced Kunming (KM) mice (Cui et al., 2022). The alleviation of cerebral I/R injury after Chry treatment may function in inhibiting NIX-mediated mitophagy.

**Xiao-Xu-Ming Decoction (XXMD)** is a classical chinese herbal medicine containing twelve herbs, including Huang-Qin (*S. baicalensis Georgi*), Fang-Feng (*Saposhnikovia divaricata (Turcz. ex Ledeb.) Schischk.*), Fang-Ji (*Stephania tetrandra S. Moore*), Ren-Shen (*P. ginseng C.A.Mey.*), Gui-Zhi (*Cinnamomum cassia (L.) J. Presl*), Xing-Ren (*Prunus armeniaca L.*), Ma- Huang (*Ephedra sinica Stapf*), Chuan-Xiong (*C. anthriscoides “Chuanxiong”*), Fu-Zi (*Aconitum carmichaeli Debeaux*), Shao-Yao (*P. lactiflora Pall.*), Gan-Cao (*Glycyrrhiza uralensis Fisch. ex DC.*) and Sheng-Jiang (*Zingiber officinale Roscoe*). XXMD has been widely used to treat stroke for thousand years in china. Studies showed its improvement of cerebral injury after ischemia and protection for neuron cells (Wu et al., 2022). Ferulic acid, zingerone, and vanillic acid in XXMD were tested to be protective for OGD/R-induced cells (Chen et al., 2022). XXMD preserved mitochondrial integrity and function via inhibiting mitophagy and reduced apoptosis via the mitochondrial p53 pathway in cerebral I/R injured rats (Lan et al., 2014; Lan et al., 2018).

The table below summarises the studies on the inhibition of mitophagy involved in cerebral I/R injury by traditional Chinese medicine (Table 2).

**TABLE 2:** Monomers/decoctions inhibiting mitophagy for cerebral IR injury.

Monomers/Decoctions	Source	*In vitro* model	*In vivo* model	Target	Chemical structure	References
Ginsenoside compound K (CK)	Ren-Shen (*Panax ginseng*)	OGD/R-induced PC12 cells	MCAO/R-induced	Mul1/Mfn2	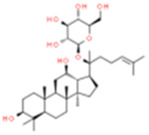	Huang et al. (2022)
			SD rats			
Rehmapicroside (Reh)	Di-Huang (*Radix Rehmanniae*)	OGD/R-induced PC12 cells	MCAO/R-induced	ONOO/Drp1/Parkin	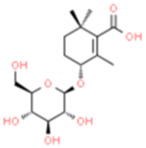	Zhang et al. (2020)
			SD rats			
Naringin	grapefruit	OGD/R-induced SH-SY5Y cells	MCAO/R-induced	ONOO/Drp1/Parkin	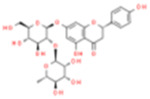	Feng et al. (2018)
			SD rats			
Artemisinin (ART)	Qing-Hao (*Artemisia annua*)	OGD/R-induced SH-SY5Y cells	——	PHB2	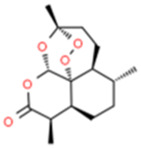	Jiang et al. (2022)
Chrysophanol (Chry)	Da-Huang (*rhubarb*)	——	CCAO/R-induced	NIX	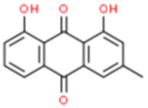	Cui et al. (2020)
			KM mice			
Xiao-Xu-Ming Decoction (XXMD)	Huang Qin (Scutellaria baicalensis Georgi), Shao Yao (Paeonia lactiflora Pall.)	——	MCAO/R-induced SD rats	——	——	Lan et al. (2018)
	Gan Cao (Glycyrrhiza uralensis Fisch. ex DC.), Fang Ji (Stephania tetrandra S.Moore)					
	Ren Shen (Panax ginseng C.A.Mey.)					
	Gui Zhi (Cinnamomum cassia (L.) J.Presl)					
	Xin Ren (Prunus armeniaca L.)					
	Ma Huang (Ephedra sinica Stapf), Chuan Xiong (Conioselinum anthriscoides)					
	Fu Zi (Aconitum carmichaeli Debeaux)					
	Fang Feng (Saposhnikovia divaricata (Turcz. ex Ledeb.) Schischk.)					
	Sheng Jiang (Zingiber officinale Roscoe)					

## 5 Discussion

Due to the limited therapeutic options available for treating ischemic stroke, researchers have increasingly focused on mitophagy as a potential target. The studies mentioned above have shown that regulating mitophagy can significally alleviate cerebral ischemia/reperfusion injury, providing *in vitro and in vivo* evidence for mitophagy as a new treatment option for ischemic stroke. mitochondrial autophagy, as well as mitochondrial fission, fusion and other processes are involved in adjusting the quantity and quality of cellular mitochondria. This further regulates the processes of cellular oxidative stress, inflammation, apoptosis, and pyroptosis, achieving a therapeutic effect and significantly alleviating neurological damage.

Currently, there are few drugs that modulate mitophagy for the treatment of cerebral ischemia/reperfusion injury. However, traditional Chinese medicine (TCM) has shown promising results as a complementary treatment for ischemic stroke in China. Furthermore, there is a substantial body of *in vivo* and *in vitro* evidence indicating that TCM can mitigate cerebral ischemia/reperfusion injury. In recent years, there has been a gradual increase in studies examining the regulation of TCM monomers and combinations.

This paper summarises *in vivo and in vitro* studies of Chinese herbal medicines that modulate mitophagy for the treatment of I/R mechanism. It was found that different Chinese herbal medicines can regulate mitophagy positively or negatively. When the organism has an excess of damaged mitochondria in situations such as ischemia and hypoxia, herbal medicines like LIG and PNS can activate mitophagy to maintain cellular homeostasis. On the other hand, when excessive mitophagy leads to a shortage of mitochondria in the organism, herbal medicines such as Reh and ART can inhibit excessive mitophagy and delay cell death. Therefore, both types of herbal medicines play a significant role in ameliorating I/R. In a clinical herbal combination for the treatment of ischemic stroke, different herbs have opposite effects on mitophagy. However, the overall therapeutic effect is clear and consistent, and may even be superior to that of the herbs alone.

Chinese medicine has a long history in China, and despite being around for thousands of years, it is still widely used due to its good clinical efficacy. Numerous *in vivo* and *in vitro* studies have provided evidence of the mechanisms through which CHM monomers work to treat diseases, but the complexity of TCM formulas has limited research to date. We think that TCM formulas have more research potential because the mechanisms of action between their various components are thought to be more complex than those of TCM monomers.

In addition, we discovered that traditional Chinese medicine regulates mitophagy to alleviate cerebral I/R inury through various pathways. The promotion of mitophagy is dominated by the PINK1/Parkin pathway, while the inhibition of mitophagy is achieved through mitochondrial fission and fusion proteins, ubiquitin-independent receptor proteins, and other pathways. After filtering, the number of studies included in this paper is limited. While the conclusion is not entirely rigorous, but it does offer some ideas for subsequent experimental validation based on the previous hypotheses.

This paper provides a brief description of the mechanisms of mitochondria and mitophagy and their roles in cerebral I/R injury. It summarises *in vivo* and *in vitro* evidence that different TCMs modulate mitophagy through different pathways, both positively and negatively, thereby alleviating I/R. The next step is to investigate how various active ingredients in the classic TCM formula for ischemic stroke work together to improve cerebral ischemia/reperfusion by maintaining mitochondrial homeostasis. This will provide new insights for the clinical development of drugs.
